# A pragmatic methodical framework for the user-centred development of an electronic process support for the sleep laboratory patients’ management

**DOI:** 10.1177/20552076221134437

**Published:** 2022-10-26

**Authors:** Maria Zerlik, Ian-C. Jung, Tony Sehr, Fabian Hennings, Christian Kamann, Moritz D. Brandt, Martin Sedlmayr, Brita Sedlmayr

**Affiliations:** 1 Institute for Medical Informatics and Biometry, Faculty of Medicine Carl Gustav Carus, Technische Universität Dresden, Germany; 2 Department of Neurology, University Hospital Carl Gustav Carus, 9169 Technische Universität Dresden, Dresden, Germany; 3 OncoRay - National Center for Radiation Research in Oncology, Faculty of Medicine Carl Gustav Carus, Technische Universität Dresden, Dresden, Germany

**Keywords:** Telemedicine, health informatics, sleep disorders, patient care management, disease management, medical informatics applications, user-centred design

## Abstract

**Objective:**

Limited capacities and ineffective care pathways result in long waiting times for patients and sporadic treatment controls in sleep medicine. As one objective of the ‘Telesleep Medicine’ project, a portal should be developed, which supports sleep specialists in an efficient and resource-saving patient management. On account of the limited project timeframe, the ‘classical’ user-centred design and evaluation methods could not be comprehensively implemented. Therefore, a pragmatic methodical framework was developed.

**Methods:**

For the iterative development of the portal, a combination of low-cost and quick-to-implement methods was used. In chronological order, these were: context interviews, personas, the development of an as-is model, a web search of design standards and good design aspects of similar systems, the development of a to-be model, the creation of an overarching mind map, and the iterative creation of mockups with simplified usability walkthroughs.

**Results:**

The feasibility of the pragmatic methodological framework for the development of a prototype for the portal was demonstrated. The used method combination resulted in a prototype based on the needs and requirements of the sleep specialists, taking into account their specific workflow and the technical implementation conditions.

**Conclusions:**

The presented pragmatic methodological framework can be a valuable resource for developers of comparable projects. The combination of methods worked well together regarding the limited timeframe and resources for concept development. For the future, we plan to implement and test the portal in the clinical field and thus enrich our framework with additional methods.

## Introduction

Sleep disorders are among the most common health complaints in the population. In Germany, 15% of the population suffers from a sleep disorder requiring treatment, for example insomnia, sleep-related breathing disorders, sleep-related movement disorders, parasomnias or hypersomnias. Untreated sleep disorders are an important risk factor for cardiovascular diseases like heart attack and stroke as well as neurodegenerative diseases like dementia.^1^ Therefore early detection, differentiation and specific treatment of sleep disorders are essential not only for patients’ quality of life but also to prevent relevant subsequent diseases.

However, the identification and differentiation of the wide spectrum of sleep disorders is a diagnostic challenge. The current diagnostic gold standard is the time-consuming cardiorespiratory polysomnography for one or two nights in a clinical sleep laboratory and examination by a sleep specialist. The usual waiting time for an examination exceeds several months due to a limited number of specialised sleep centres and often ineffective treatment pathways regarding follow-up visits and treatment controls.^2^ After diagnosis and initiation of treatment, many patients do not become active if therapy problems occur or, under certain circumstances, do not notice them. Therefore, follow-up visits after a few weeks or month are recommended. As a consequence of strictly fixed re-examination intervals that do not take into account the individual course of the disease, some examinations will unnecessarily tie up sleep centres capacity while others will be delayed. On the other hand, continuous tracking of individual treatment success by the sleep specialist consumes time and personal resources.

Medical and technical innovations offer the potential to counteract these problems. However, corresponding implementations that cover the entire care process of patients with sleep disorders (screening – diagnostics – therapy adjustment – follow-up) are not yet known for sleep laboratories in Germany. For this reason, a subproject of ‘Telesleep Medicine’ (funded by the European Regional Development Fund – EFRE)^3^ was started. It aims to develop appropriate structures for the telemonitoring of sleep medicine data in the home environment in order to be able to intervene proactively and early, to improve the decision-making basis for patients with doubtful findings, to reduce the control appointments per patient and thus to increase the capacities in the sleep laboratory. In addition, an electronic process support portal (hereinafter referred to only as ‘the portal’) will be developed to enable resource-efficient patient management in the sleep laboratory. This includes the support of the screening process of patients for the decision of admission to the sleep laboratory, the structured documentation of the anamnesis interview as well as the diagnoses and decisions, the support of the interpretation of the sleep recordings and automatic physician letter generation. At the same time, the portal should also offer the possibility for patients to communicate electronically with the physicians of the sleep laboratory and to support the further treatment course.

The presented paper focuses on the development of this portal for the sleep laboratory at the Interdisciplinary Sleep Centre of the University Hospital Dresden. The sleep laboratory has three polysomnography places for inpatient investigations. Further investigation and treatment control devices exist for outpatient examinations. Over 700 polysomnographies are performed per year, and patients from the entire spectrum of sleep disorders are treated. The medical care in the sleep laboratory is provided by a team of two sleep specialists with additional qualifications in sleep medicine and one to two physicians in training. The development of the portal should meet the following project requirements: embedding in the respective work environment and support of the specific processes in the sleep laboratory, intuitive, user-friendly interface, realisation of the development and implementation within a project period of 13 months (November 2020–December 2021), technical feasibility under special requirements of integration/data exchange possibilities at the University Hospital Dresden and, if possible, use of already existing systems, tools and data exchange possibilities.

For the development of clinical applications and systems, user-centred design (UCD) can be used as an established process model.^4^ Nevertheless, this requires about 5–10 iterations until an appropriate solution emerges that is highly usable.^5^ UCD methods are rather costly and cost more time in the development process, even if they ‘pay off’ later by minimising rework efforts.^6,7^ However, for development projects with limited time, limited human resources, and the difficulty of obtaining test subjects in a clinical setting, a more pragmatic methodological approach is needed. Although discount usability methods have been increasingly published in recent years,^8,9^ there is no real selection support in the literature, which methods can be combined best and under which general conditions. Furthermore, there is no template of a possible combination for the medical field and thus no support for the selection, application and modification of possible methods for the development of portals for sleep medicine.

That is why the paper presents an approach with a set of low-cost, quick-to-apply and fast-result methods and describes a pragmatic methodological framework for the user-centred development of an electronic process support for the sleep laboratory patients’ management. The aim of this paper is to provide a blueprint, which can be used by other researchers and developers for comparable projects.

## Methods

For the user-centred development of the portal for the management of patients in the sleep laboratory, the UCD with its four phases of context analysis, requirements definition, solution development, and evaluation of solutions against user requirements was used as basis.^4^ For each of these four phases, appropriate methods were selected that meet the specific constraints of the project (limited time, limited human resources, limited availability of medical staff for design input and evaluations, need for rapid & feasible results). Table 1 provides an overview of the method set used, which is described in more detail below. The project team consisted of one somnologist, two medical technologists, two sleep physicians, three research assistants with usability expertise and three medical informaticians.

**Table 1.** Methodological framework for the user-centred conceptualisation of the portal in chronological order.

UCD phase	Method name	Brief description of implementation	Main result	Persons needed	Material needed	Total time required (full-time days)
**Context analysis** (UCD phase 1)	Persona development	Based on literature analyses of existing statistics and unstructured brief interviews of end users; feedback round for validation (via email)	Persona for each user group as a PowerPoint slide	Literature analysis: 1 researcher Interviews: 2 end users Persona development: 1 researcher Feedback round for validation: 2 end users	Paper, pen, online access, presentation software (e.g. Microsoft PowerPoint), meeting room	2.5 days
	Context interviews	On-site observation and interview of an end user based on a prepared set of questions (e.g. tasks/subtasks sequence, person performing, materials needed), tabular documentation of interview results; feedback round for validation (via email)	Tabular task/work process description	Context interviews: 1 end user, 3 observers/interviewers Feedback round for validation: 2 end users	Paper, pen, writing programme (e.g. Microsoft Word)	3 days
	Visualisation of ‘as-is processes’ (without system)	Modelling of the current work processes using a simplified ‘Business Process Model and Notation (BPMN)’ language and text annotation; feedback round for validation (via email)	Model of existing work processes	Creation of models: 2 researchers Feedback round for validation: 2 end users	Modelling software (e.g. open-source modelling tool Camunda Modeler)	4 days (depending of the complexity of the work processes)
**Requirements definition** (UCD phase 2)	Derivation of initial usage requirements from context information	Screening of context interview documentation for user needs, formulating requirements according to the template: ‘The user must be able to enter/select/recognise/overview “X” (=designation of the type of the information) in the portal.’; feedback round for validation and prioritisation of requirements (via online meeting/conference)	Prioritised usage requirements	Derivation and definition of usage requirements: 1 researcher Feedback round for validation and prioritisation: 1 moderator, 2 min-takers, 2 end users	Presentation as the basis for discussion (e.g. in Microsoft PowerPoint), communication software for online meeting (e.g. Zoom), paper and pen or documentation/writing programme (e.g. Microsoft Word)	1.5 days
**Iterative solution development with interim evaluations** (UCD phases 3 and 4)	Market analysis	Internet research (Google) of similar systems (competitors) and tabulated documentation of: product name, link to the product website, promising design decisions (which could be used as an inspiration for the project prototype), controversial design decisions (which should be avoided for the project prototype) and screenshots of the systems (if publicly accessible)	List of potential design features to be included and excluded	1 researcher	Online access, documentation/writing programme (e.g. Microsoft Word)	2 days
	Guideline collection	Internet research (PubMed) of possible design guidelines/norms/standards; screening of the international standard series DIN EN ISO 9241: Ergonomics of human–system interaction for relevant guidelines	List of (ergonomic) recommendations to be considered for the design	1 researcher	Online access, access to DIN EN ISO Norms, documentation/writing programme (e.g. Microsoft Word)	2 days
	Visualisation of ‘to-be processes’ (with system)	Modelling of the future work processes using a simplified ‘Business Process Model and Notation (BPMN)’; feedback round for validation (via email)	Model of the envisioned future work processes	Creation of models: 1 researcher Feedback round for validation: 2 end users	Model of existing work processes, modelling software (e.g. open-source modelling tool Camunda Modeler)	2 days
	Development of an overall screen concept	Creation of a mind map, which illustrates the various areas of use for the target groups with the functions and information required in each case; feedback round for validation (via stakeholder online meeting)	Overarching screen concept	Creation of the mind map: 1 researcher Feedback round for validation: project team	Mind map software (e.g. XMind), Communication software for online meeting (e.g. Zoom)	1.5 days
*(1st iteration)*	Development of a wireflow concept	Discussions of technical feasibility (via stakeholder online meetings); marking of screen areas that are feasible for implementation in the overarching concept/mind map; conversation of the overarching concept/mind map into a wireflow using Balsamiq and yEd	Screen flow illustration	Discussion of technical feasibility: project team Creation of wireflow: 2 researchers	Communication software for online meeting (e.g. Zoom), wireflow tools (e.g. Balsamiq with yED)	4 days
	Evaluation and refinement of the wireflow concept	Interview of an usability expert: presentation of the concept and documentation as well as incorporation of the feedback into the wireframe concept Stakeholder online meeting: presentation in front of the project team and documentation of the changes Workshop with end users: presentation and collection of changes	Evaluated wireflow concept	Expert interview: 1 moderator, 1 min-taker, 1 usability expert Stakeholder meeting: project team Workshop: 1 moderator, 1 min-taker, 1 end user	Presentation of the wireflow concept (e.g. via PDF or print), communication software for online meeting (e.g. Zoom), pen and paper for documentation	3.5 days (expert interview: 1 day, stakeholder meeting: 0.5 days, workshop: 2 days)
*(2nd iteration)*	Development of a low-fidelity prototype	Conversion of the wireflow into a low-fidelity prototype using Balsamiq; implementation of the changes suggested in the evaluation of the wireflow concept	Low-fidelity prototype	2 researchers	Mockup tools (e.g. Balsamiq with yED)	4 days
	Evaluation of the low-fidelity prototype	Usability walkthrough with a usability expert: presentation of the prototype and documentation of the changes suggested; usability walkthrough with 2 end users	Evaluated low-fidelity prototype	1 moderator, 1 min-taker, 1 usability expert, 2 end users	Presentation of the prototype (e.g. via Balsamiq), pen and paper for documentation, meeting room	2 days
*(3rd iteration)*	Development of a medium-fidelity prototype	Development of a clickable mid-fidelity prototype based on an average fictitious patient and suggested changes of the evaluation of the low-fidelity prototype	Medium-fidelity prototype	2 researchers	Wireflow tools (e.g. Balsamiq with yED)	4 days
	Evaluation of the medium-fidelity prototype	Online walkthrough with end users based on typical work tasks; guiding of end users section per section on the basis of prepared evaluation questions; documentation of suggestions for changes, end user rating of satisfaction and further needs for interface revisions	Evaluated medium-fidelity prototype (=‘design freeze’ for implementation)	1 moderator, 2 min-takers, 2 end users	Presentation of the prototype (e.g. via Balsamiq), communication software for online meeting (e.g. Zoom), pen and paper for documentation	1.5 days

*Note*: In the demonstrated use case, the end users included in the user-centred design were sleep specialists. The absolute time required for each step includes preparation, execution of the method(s) and evaluation of the results.

### Context analysis: analysis of user, tasks and work environment

At the beginning of the portal development, the primary (physicians), secondary (nurses) and tertiary (patients) user group of the portal was defined during a project meeting as a consensus decision of the project team. Subsequently, user characteristics for these target groups were collected on the basis of Internet research (statistics on the target groups, for example, average age, expertise, etc. from the websites of the Federal Statistical Office and the German Medical Association) and interviews of two sleep specialists during a stakeholder meeting. Missing information was added as assumptions/hypotheses. The tabularised information for each target group included user group, age range, gender, language, work experience/qualifications, physical limitations, prior experience with similar systems, computer experience, and in relation to the portal: motivation to use and fears & concerns. With the help of this tabular information, so-called personas^10,11^ (=user models characterising persons of a target group in their characteristics) were developed and reviewed by two sleep specialists.

To record the current work processes, a sleep physician was interviewed and observed in the form of a context interview, working in the field by three research assistants. Interview questions referred to the (typical) task sequence and the work results as well as the persons involved (physician, nurse, patient) and the materials used. The interview and observation results were structured in tabular form (table columns: main activity, partial activity, persons involved, location and material). The afterwards summarised protocols were then reviewed by sleep specialists for correctness and completeness. The tabular information was converted into a graphical specification language using the Business Process Model and Notation (BPMN) (https://www.bpmn.org/), which provides symbols to model and document workflows. To simplify communication with sleep specialists, we decided to limit the visualisation to the following BPMN objects or symbols: pool, lane, event (none start/end event, message and timer), task, gateway (parallel or exclusive), message, data object, data store and text annotation. The used visualisation tool was the open-source modelling tool Camunda Modeler (https://camunda.com). The visualisation was again reviewed by two sleep specialists of the sleep laboratory.

### Requirements definition

The context interviews were screened with regard to explicitly stated and implicit requirements and wishes for a portal design. From this initial usage, requirements were formulated according to the pattern ‘The user must be able to enter / select / recognise / overview “X” ( = designation of the type of the information) in the portal’, which were structured according to the core tasks. The requirements were then reviewed for completeness and correctness during an online meeting with two sleep specialists and prioritised (priority 1 = high, to be implemented in the project, priority 2 = medium, if there is still time and resources, priority 3 = rather to be considered for future work).

### Iterative development of solutions including evaluations from the user's perspective

#### Market analysis and guideline collection

For the identification of design ideas, a market analysis was carried out by performing a variety of Google (https://www.google.de) searches. As a result of the analysis, the product name, link to the product website, promising design decisions (which could be used as an inspiration for the project prototype), controversial design decisions (which should be avoided for the project prototype) and screenshots of the systems (if publicly accessible) were tabulated. In addition, existing usability guidelines were searched in the Database ‘PubMed’ (https://pubmed.ncbi.nlm.nih.gov/) with the terms guidelines, recommendation, usability, principles and best practices in combination with the terms telemedicine, telehealth or remote patient monitoring. Additionally, the international standard series DIN EN ISO 9241: Ergonomics of human–system interaction^12^ was scanned for its relevance for the project scope. As a result, a reference table of the possible relevant norms was created, which served as a base for the design decisions during the development of the prototype.

#### Modelling of future work processes with portal

Following the derivation of user requirements, a target process that illustrates the future work processes with the portal was developed. For this purpose, the BPMN visualisations of the actual processes were modified or replaced by the modified processes under portal use. The target processes were reviewed by sleep specialists with regard to reasonableness and completeness and revised again based on this feedback.

#### Development of an overarching screen concept

Based on the to-be model of processes and initial usage requirements, functions were derived by a researcher with usability expertise and a comprehensive screen concept was developed in the form of a mind map using XMind (https://www.xmind.net). This illustrated the various areas of use for the target groups with the functions and information required in each case. The mind map was reviewed during a stakeholder online meeting and revised on the received feedback.

#### Iterative development and evaluation of a mockup prototype

Subsequently, a mockup prototype of the portal was iteratively developed in three phases. For this purpose, the developed mind map was discussed beforehand in terms of technical feasibility in various stakeholder meetings with a somnologist, sleep physicians, medical technologists, scientists with usability expertise and medical informaticians. The screen areas that were feasible for implementation were then marked accordingly in the mind map.

In the first phase, the mind map was converted to a wireflow. Different paper and pencil versions were created, which were then aggregated into one electronic version, using Balsamiq (https://balsamiq.com/wireframes/desktop/) and yEd (http://www.yWorks.com). The developed wireflow was then evaluated and further refined in several rounds: (1) evaluation by interviewing a usability expert in the course of a one-hour interview and revision of the wireflow, (2) presentation of the improved version in a virtual stakeholder meeting (web conference) and documentation of the suggestions for improvement and (3) conducting a workshop with a sleep specialist using a printed version of the wireflow presented on a whiteboard and documenting the suggestions for change.

In the second phase, the wireflow was converted to a low-fidelity prototype in Balsamiq. For this purpose, the suggested changes were implemented. The low-fidelity prototype was then evaluated separately with a usability expert and two sleep specialists during usability walkthroughs, demonstrating the user flow of the prototype and documenting corresponding suggestions for change.

In the third and final phase, the suggested changes from phase two were implemented and a clickable medium-fidelity prototype was developed based on an average fictitious patient of the interdisciplinary sleep laboratory at the University Hospital Dresden. The medium-fidelity prototype was then evaluated in an online walkthrough via Zoom (https://zoom.us) on the basis of typical work tasks by two sleep specialists. For this purpose, the sleep specialists were guided through the prototype section by section, whereby they were asked to indicate, on the basis of prepared questions, where they would assume certain information to be located and which further interaction steps they would take. At the end of the walkthrough, the sleep specialists were asked to rate their overall impression of the prototype on a 6-point rating scale from ‘very good’-1 to ‘insufficient’-6. In addition, the sleep specialists were asked to rate on a 10-point rating scale from ‘nothing’-0 needed to be changed to ‘a great amount’-10 how much additional development work would be necessary regarding the design so that it would meet their wants and needs. The importance of the identified issues and the improvement suggestions for their impact on the project was then assessed by the project team and changes were made for the most important issues.

## Results

In the following, we present the main results of our work using the method combination described above, which also demonstrates the applicability of our pragmatic framework.

### Results of the context analysis

The target groups of the portal were defined as follows: physician for sleep medicine – primary user, nurse in sleep laboratory – secondary user, and patient with sleep disorders – tertiary user. A summary of the main user characteristics can be found in Appendix 1, supplementary material; the type of information collection (stakeholder meeting, literature analysis, project assumptions) was marked in each case. Based on the user characteristics collected, personas were developed for each target group, which are presented in Appendix 2, supplementary material.

The current work processes (without portal) were visualised using simplified BPMN notation. The processes were divided into three sub-processes: preclinical, clinical and postclinical. For illustrative purposes, only the clinical main processes are presented here (a detailed description of all processes can be requested from the authors if required). The usual process flow is as follows: ‘On the day of the sleep laboratory visit, the patient first registers centrally at the hospital, and is given a patient ID and case number. The patient then proceeds to the sleep laboratory. After the patient is admitted, the nurse records the patient's data (e.g. height, weight, blood pressure), asks for further important information (e.g. care needs) and documents this information in the paper patient record. The patient then receives paper questionnaires on sleep disorders and is asked to complete them. The admission data is then transferred to the hospital workplace system and completed patient questionnaires are scanned in. The physician then takes the patient's medical history and documents complaints, results of the physical examination, previous findings, comorbidities and medication as well as suspected sleep diagnosis on the basis of examination, interview and questionnaires in the hospital workplace system. For the actual sleep examination (polysomnography), the patient is then connected to various sensors for sleep recordings. The following biosignals are measured during the whole night: electroencephalogram, eye movements, muscle tone from the chin, respiratory flow, electrocardiogram, leg movements, respiratory movements of the chest and abdomen, oxygen saturation, body position, activity (video) and snoring. The sleep recording is monitored by sleep laboratory staff during the night and then evaluated by a trained medical technical assistant using the hospital workstation system the next morning. For this purpose, recordings are manually annotated and colour-coded according to a standardised set of rules by the American Academy of Sleep Medicine (AASM), and an electronic PDF report with all important values is then generated. The physician checks the evaluation of the polysomnography and the report, interprets the values and then makes a confirmed diagnosis, which in turn is documented electronically in the hospital workplace system. The physician then discusses the evaluation of the sleep recording and possible therapy options with the patient. On the second day in the sleep laboratory, the patient receives an appropriate therapy (e.g. positive airway pressure device or medication) and the treatment effects are monitored during sleep. The sleep recording is evaluated by the medical technical assistant as already described above. After interpreting the sleep report and discussing the results with the patient, the physician writes a preliminary report including patient history, results, interpretation and treatment recommendations. If necessary, an aid prescription or a prescription for billing of technical devices to the health insurance company will be issued.’

**Figure 1.**
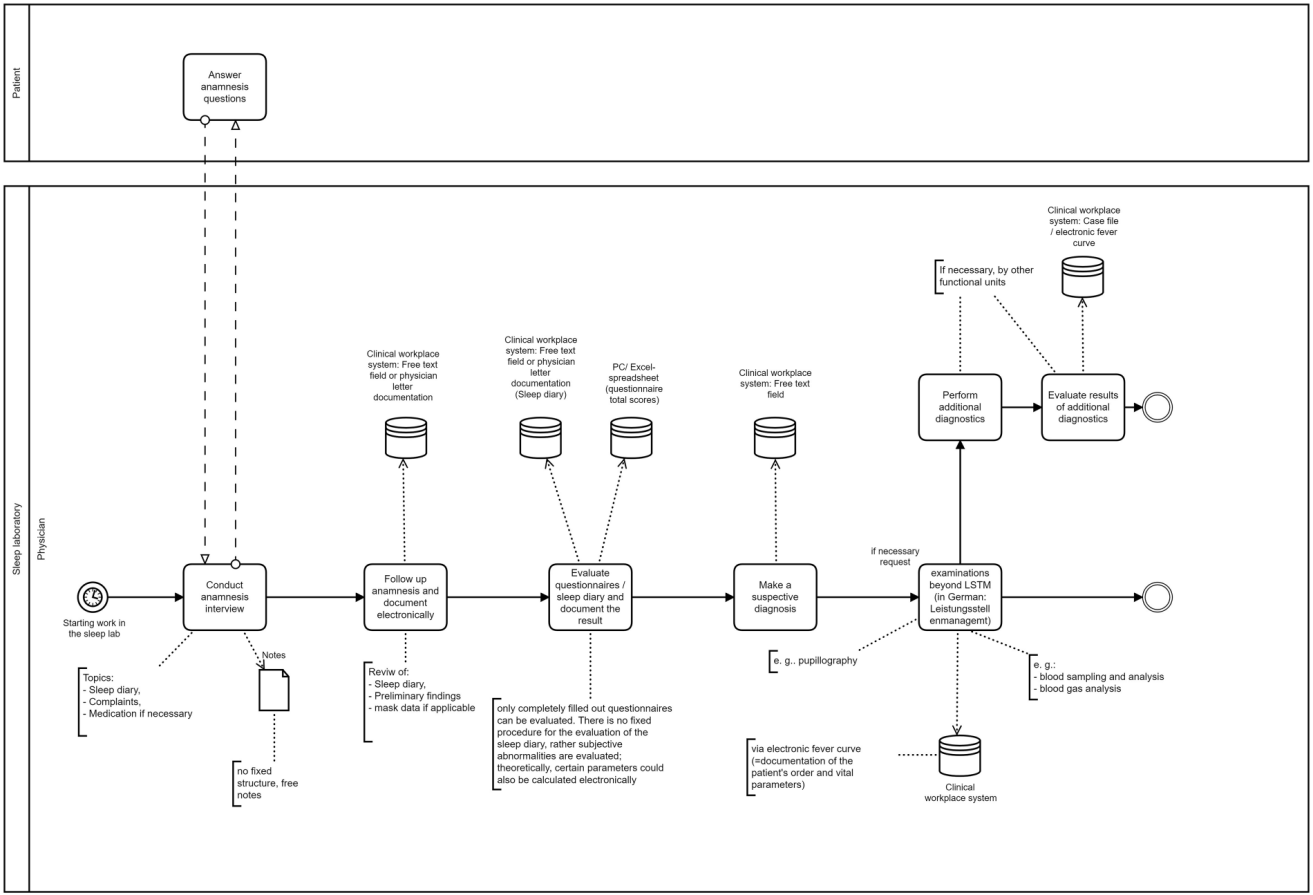
Excerpt from the BPMN as-is model (clinical phase – day 1).

Figure 1 shows a section of the corresponding visualisation for the process phase ‘clinical (day 1)’.

### Results of the requirements definition

A total of 33 initial usage requirements were derived from the results of the context interviews, of which 21 requirements were rated as ‘highly relevant – essential to implement within the scope of the project (prioritisation 1)’ (see Appendix 3, supplementary material).

### Results of the iterative development of solutions

#### Market analysis and guideline collection

In the market analysis, three telehealth platforms,^13–15^ two e-health physician/patient portals^16,17^ and two sleep laboratory management systems^18,19^ were analysed. Positive design aspects from an ergonomic point of view, which were selected as possible design ideas, were as follows:

- Patient data such as medication list, latest diagnoses, latest reports, executed treatments, most recent laboratory results and vital signs summarised on one screen
- Use of tabs for each patient for comfortable switching between different patient records
- Highlighting need for action by red warning symbol in the patient list
- Display of treatment steps by status bar
- Search function: finding patients by name or other parameters
- Optical highlighting of important laboratory values
- Presentation of hospitalisation by using timeline

Negative design aspects, which should be avoided from an ergonomic point of view in the prototype were:

- Use of more than two menus bars (leads to longer orientation times for the user or makes it more difficult to recognise the navigation clearly and quickly)
- Display of patient's contact information in a prominent spot on the user interface (information that is not important for the current action should not be placed in the user's field of vision).

The compiled usability guideline collection includes recommendations for documentation and diagnostic interfaces,^20^ visual information presentation,^21^ form dialogue design,^22^ user interface elements,^23^ web user interfaces^24^ and user-centric security and data privacy aspects for telemedicine services,^25^ which is shown in Appendix 4, supplementary material.

#### Modelling of future work processes with portal

Based on the usage requirements and the BPMN as-is model, a BPMN to-be model was developed in which the processes supported by the portal were marked or added (if new processes).

**Figure 2.**
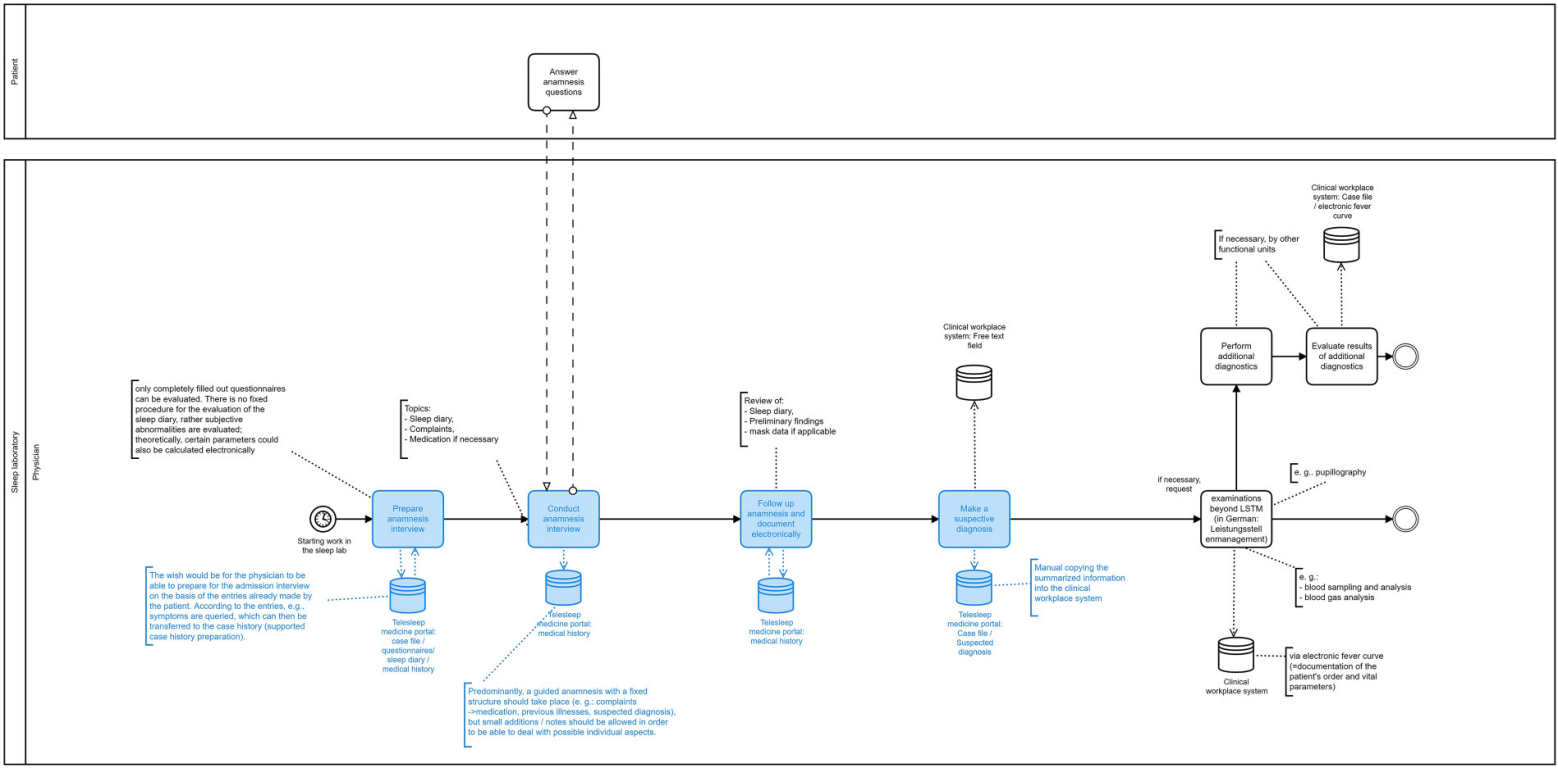
Excerpt from the BPMN to-be model with portal (clinical phase – day 1). The future processes with portal are highlighted in blue.

Figure 2 shows a section of the visualisation of the to-be model for the clinical phase associated with the as-is model in Figure 1.

In relation to the clinical phases, the following process steps should be supported by the portal:

- Electronically assisted admission and collection of medical history data (nursing staff)
- ‘Handing over’ electronic sleep history and sleep disorder questionnaires to patient (physician)
- Completion of electronic questionnaires (patient)
- Electronically supported preparation, execution, follow-up and documentation of the anamnesis interview (physician)
- Electronically supported establishment of the suspected diagnosis (physician)
- Electronically supported assessment and printing of the sleep report (physician)
- Documentation of the diagnosis or the evaluation of the therapy success and further procedure (physician)
- Writing and sending of the doctor's letter and if necessary the prescription (physician)

#### Development of an overarching screen concept

**Figure 3.**
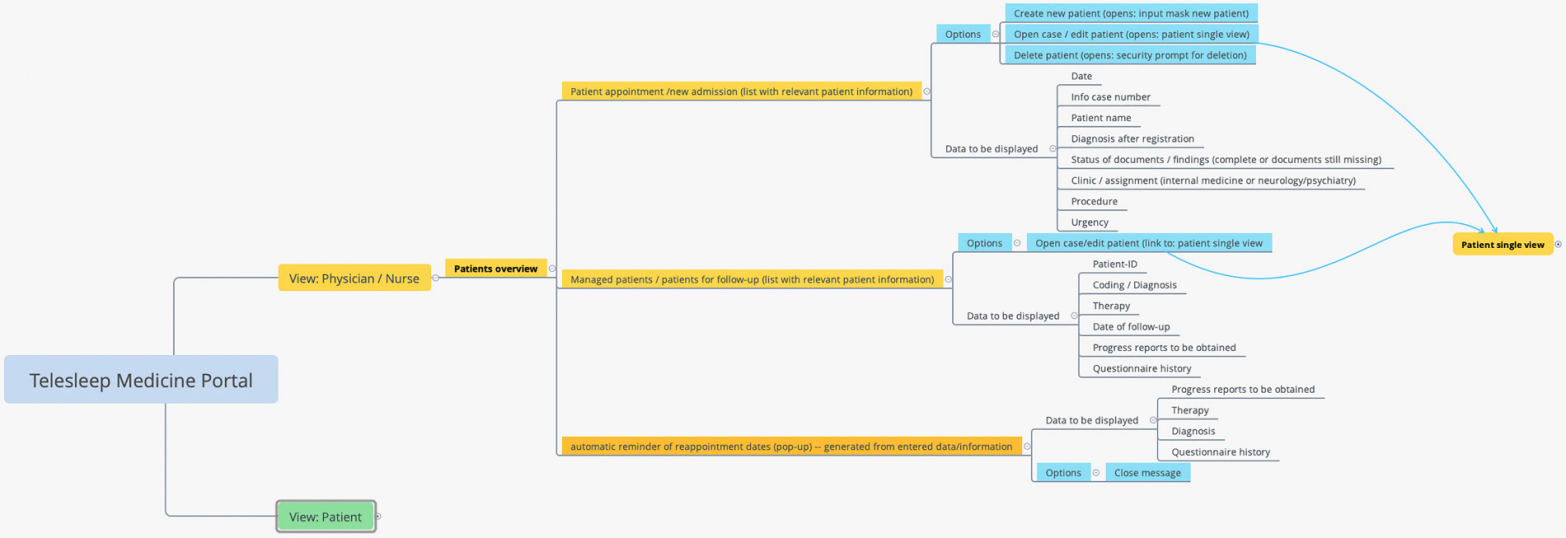
Section of the overarching screen concept (implemented with XMind) for the user group physician.

To illustrate the overall screen concept, a corresponding mind map was created. Figure 3 shows a section of the mind map. The common presentation of functions for physicians and nurses is due to the fact that there are no separate areas of the system for these user groups, that is, both user groups see the same user interface (but use different functions of the interface for certain tasks).

#### Iterative development and evaluation of a mockup prototype

**Figure 4.**
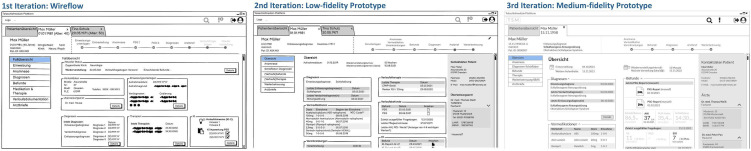
Iterative interface concept development (based on the ‘single patient – overview’ screen). Excerpts from (left to right): wireflow, low-fidelity prototype and medium-fidelity prototype.

The mockup prototype was developed as a wireflow and under the following identified project-specific constraints: Usage of already existing systems, tools and interfaces/communication possibilities with existing systems of the University Hospital Dresden, restriction of the implementation to the treatment process starting from ‘patient in the sleep laboratory’/clinical phase, resource-related restriction to the physician's portal view, and focus on the display of data/visualisation of existing information. Based on these constraints, the interface was conceptualised. Figure 4 shows the three iterations based on the ‘single patient view – overview’ screen.

The developed wireflow included the following main functions:

- Patient overview (table with all patients) with search, reminder and filter function
- Tab for each single patient view
- Display of status bar to check the steps performed in the treatment process of an individual patient.

After evaluating the wireflow, the contents of the individual screens were created in detail. The following additions and changes were made for the low-fidelity prototype:

- Determination of the structure for all tables (table columns)
- Display of the polysomnography (PSG) report & neurology questionnaire scores
- Adjustment of the terminology
- Adjustment of the status bar
- Comparison of manual and AI-based PSG reports: Switching on the AI-based PSG report by toggle button

For the medium-fidelity prototype, the tables and text fields were filled with exemplary patient data. The following changes and additions were made after evaluating the low-fidelity prototype:

- Adjustment of filters on the ‘patient overview’ screen
- Addition of favourite function: Users can mark patients as favourites, which then are displayed in list of favourites
- Display of important questionnaire scores and PSG values depending on discharge diagnosis (on single patient view – overview)
- Display of short PSG report and full PSG report
- Display of trend for two PSG reports

**Figure 5.**
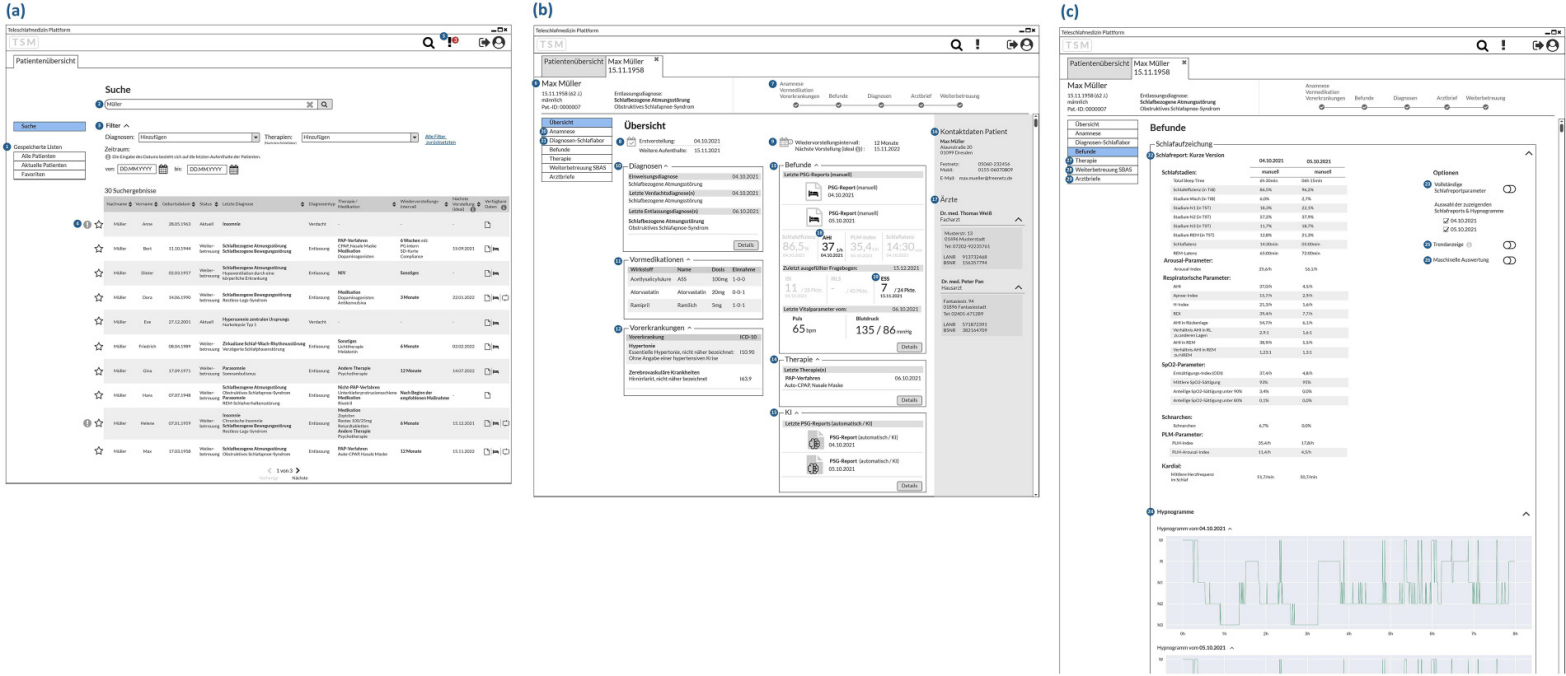
Excerpt from the medium-fidelity prototype: (a) ‘patient overview – search’, (b) ‘single patient view – overview’ and (c) ‘single patient view – findings’.

Finally, the medium-fidelity prototype was evaluated with two sleep specialists. The complete collection of all prototype screens is available in Appendix 5, supplementary material. In the following, the main functions and information fields of the prototype are described:

After users have logged in via the landing page, they reach the ‘patient overview’ home screen which is shown in Figure 5 (a).

Via the page menu users have the opportunity to choose from different kinds of saved patients’ lists (1): patients present in the system (‘All Patients’), patients present in the sleep laboratory (‘Current Patients’) and star-marked patients (‘Favourites’). Users can search for a specific patient by name (2) and filter (3) like diagnosis, therapy and time period, which refers to the patient's last stay. The results of the search are presented in a table. By using the exclamation marks in the table (4) and in the header (5), users can check which patients are due for their ideal calculated follow-up appointment in the next two weeks.

Clicking on a table row opens a new tab, which presents the single patient view (see Figure 5(b)). The tab header displays key information such as date of birth, age, gender, patient ID and discharge diagnosis (6). Furthermore, in the tab header, users can check which steps in the treatment process have already been performed using the status bar (7). The tab header is permanently visible when using the single patient view. On the overview page of the single patient view, users can inspect the patient's appointment history for initial presentations and further stays (8). Also, the re-presentation interval and the calculated ideal re-presentation date are displayed (9). The overview page presents the most recent sleep laboratory diagnoses (10), prior medications (11), pre-existing conditions (12), findings (13), most recent therapies performed (14), availability of AI-generated PSG reports (15), patient contact information (16) and patient's treating physicians (17). By using the findings tile, users can review the most important values from the PSG report (18) and questionnaire scores (19) depending on the discharge diagnosis. Less important values for the discharge diagnosis are shown in light grey. In the menu item ‘Anamnesis’, users can view the nine most recently collected questionnaire scores and the questionnaire-based, automatically generated anamnesis text (20). By using the menu item ‘Diagnoses Sleep Lab’ (21), users can list all sleep laboratory initial, tentative and discharge diagnoses.

Users can find the existing short PSG report (22) and complete PSG reports^23^ with the corresponding hypnograms (24) in the menu item ‘Findings’ (Figure 5(c)). A toggle button can be used to inspect the trend of two PSG reports by showing it in an additional column (25) Users can use another toggle button to view the AI-generated PSG reports with quality criteria of the AI-based evaluation (26) and compare them with the manual PSG reports. The findings page shows the graphical progression of the questionnaire scores by unfolding the table as well as vital signs such as height, weight and BMI. Therapies performed in the sleep laboratory or after the sleep laboratory can be displayed via the menu item ‘Therapy’ (27). The page ‘Continuing Care Sleep-related breathing disorder (in German: Schlafbezogene Atmungsstörung (SBAS))’ presents the therapy compliance and effectiveness, which was rated by the physician as well as the patient (28). Also, the average apnea-hypopnea index and the average duration of use of the continuous positive airway pressure mask are also displayed. The automatically generated draft of the physician's letter can be obtained via the menu item ‘Physician's letters’ (29).

The evaluation of the medium-fidelity prototype showed that most functions were found directly by the physicians. If features were not immediately discovered, they were considered useful and important after being presented. The ‘single patient view – overview’ was perceived to be particularly well designed (quote: ‘I would wish to see that for our patient record system’). In addition, the PSG-report representation and the trend display was experienced to be very refinded and helpful (quote: ‘significant improvement over the current view’, ‘very cool’).

Both sleep specialists rated their overall impression of medium-fidelity prototype as ‘good’ (rating of 2 on a 6-point scale) with a tendency to ‘very good’ (rating of 1). Additionally, they estimated that, when considering the project limitations, very little work (rating from 1–2 on a 10-point scale) would still be needed on the prototype design. Final improvement suggestions to be considered for later implementation were: In the ‘patient overview’ page, the column patient ID should be added to the table. Furthermore, there should be the possibility to search by patient ID. As it turns out, some physicians prefer to use last name and date of birth, some use the patient ID to access the patient's record. In the medium-fidelity prototype on the patient overview page, the time period relates to the last stays of the patients. For further development, there should be the possibility to set the reference period to the last stays and the first stays of the patients. In the presentation of hypnograms, attention should be paid to the existing specific graphical representation standards for different sleep phases. The short and the full PSG-report have to be checked for completeness, because during the evaluation the value ‘minimum oxygen saturation during sleep’ was missing in the short PSG report.

## Discussion

The goal of our work was the provision of a set of user-centred methods in the form of a pragmatic framework for rapid user-centred development of a portal concept and interfaces for the management of patients with sleep disorders with few time and human resources. For the user-centred development of the portal, we followed the UCD design process, which has already been proven to be effective.^26,27^ We chose a combination of low-cost, quick-to-implement methods^28,29^ and modified them for the clinical context and our project conditions, respectively, to develop our portal in a user-centred way. Interim evaluations of drafts have generally required only a few hours, including preparation and evaluation of no more than 2 days (per individual method). In addition, evaluations did not require a fully functional, implemented prototype or expensive equipment, and just a few subjects (two sleep medicine specialists, one usability expert, members of the project team) were enough to identify important needs for change. Our framework worked well and proved to be effective in constantly improving the design of the portal over only a few cycles. In addition, the methods applied have enabled us to foster communication in the project, especially between developers and sleep specialists, and to create a common vision of the end product ‘portal’, which was perceived as very profitable by all sides.

As far as studies of similar development/portals for sleep laboratories are concerned, the possibilities for comparison of appropriate approaches are very limited: While there are numerous studies addressing portals for patient management, these focus more on physician-patient communication, benefits and outcomes, and other application areas.^30–32^ Related to sleep disorders and patient management process support for physicians, we could identify only two relevant studies that address a user-centred development:

Pulantara et al.^33^ describe the system iREST, which had the goal of better connecting patients and clinicians. With the implementation of electronic sleep diaries and patient questionnaires, automatic data collection, and progress visualisation for the clinician, the same functions were pursued as with our sleep medicine portal. The researchers also used an iterative and incremental development approach and derived requirements from current work processes. However, these requirements describe desired functionalities and requirements for the quality in which the required functionality is to be provided (e.g. real-time communication, privacy and security) and do not represent true usage requirements derived from user needs (as for example in our case: ‘The user must be able to obtain information about the patient in the portal based on information already entered’). The work also refrains from describing user characteristics, considering ergonomic principles for interface design, and using mockups and preview prototypes to get feedback for design changes even before (more elaborate) implementation. In comparison, however, our framework offers methods that put the user with his specific work tasks and his specific usage environment in the centre of the work at any time of the development process to avoid that developments are too technology-focused.

The second study by Blake et al.^34^ describes a sleep tool web application to assist sleep specialists in making diagnoses. The authors’ project, like ours, aimed to improve communication and workflow by redesigning a complex manual process with technological support. A Design Science Research approach was used as the framework model for the development, which develops solutions oriented to the goals of the users. The approach described also uses a mix of different user-centred methods, but, compared to our methodological approach, focuses very much on the design artefact without a comprehensive analysis of the context of use, and only roughly defines user roles without precisely detailing the specific user characteristics and specific work tasks in each case. This carries the risk that person-related, task-related or environment-related factors that have an important influence on usage are not or insufficiently included in the design.

Compared to both papers, our methodological framework supports the consideration of specific user characteristics and work processes to enable a fit of the portal to the specific user needs. We, therefore, believe that our framework can be a valuable complement to existing approaches and could serve researchers and developers as a blueprint for comparable projects in combining specific user-centred methods. With regard to the results of the framework, our interface concept can be a good source of design ideas; likewise, the design principles and standards we researched can be a good input for the development of corresponding solutions of other sleep laboratories. The personas with their characteristics are also reusable for other German sleep laboratories.

In closing, we would like to share the following lessons learnt:

- A visualisation of the work processes (as-is situation) by using a simplified BPMN notation turned out to be very beneficial to be able to see the sequence and dependencies of the processes well at a glance. For sleep specialists, this mapping was quick and easy to understand and made communication within the project team much easier. In retrospect, we think it would have been better not to try to map the entire process in detail right away, but to start at a fairly rough level. Detailed modelling could then be carried out only for the focus area, so that additional time and resources could be saved here.
- A mapping of the required areas/views and functions of the portal via a mind map (using XMind) clarified the complexity of the project well for the project partners and led to the fact that, for the first time, the different ideas of the project goal (developers and physicians each had a different idea of what the ‘portal’ should be) would also be discussed.
- The three iterations for developing an interface concept proved to be a very good strategy. However, with hindsight, the creation of the wireflow (1st iteration) was a bit too detailed from our point of view, as we found out during the evaluation. The focus of this evaluation was on the rough menu structure, the logic of the operating sequence or navigation and the completeness of the functions. However, due to the detailed design, the interviewees were tempted to keep giving feedback regarding the information presentation and design, but this was only relevant in later iterations.
- For the medium-fidelity prototype, we also used Balsamiq for reasons of not having to learn another separate tool, but it quickly reached its limits for this type of prototype: dynamic changes to the page content could only be implemented very cumbersomely by copying and changing the pages several times. Also, changes had to be made for all copies. In addition, tables could only be adapted to a limited extent and fine adjustments were tedious. Since the saved learning time was consumed by the additional effort during prototyping, we would generally recommend other tools such as Mockplus (www.mockplus.com), Axure (www.axure.com) or Justinmind (www.justinmind.com) for medium-fidelity prototypes.

### Limitations

Our intention was to show developers of similar systems an implementable methodical way how to design their applications user-centred, that is, with very early involvement of the users in the development work. Although we defined all user groups at the beginning, we limited ourselves in the later presentation to the implementation of the interface for the physician. However, we think that the framework is just as applicable for nurses and patients as the literature shows^35^ that individual methods have already been carried out with these user groups (albeit in other application areas).

By using evaluation methods with only a few sleep specialists, we were able to identify important usability issues that gradually improved the design. However, in-depth usability analyses (e.g. click times, exact analysis of user behaviour or psychological patterns) were not possible with these methods. Yet our pragmatic framework does not claim to replace classical, traditional usability testing and other usability engineering methods. Rather, it should be a motivation to overcome the ‘usability testing’ barriers in the first place, and not to seek interim feedback only at the end, when the system is already implemented, because ‘testing is costly’. Another limitation is that we have not evaluated our framework quantitatively. For example, it would have been conceivable to ask the project participants again more specifically at the end of the development how they rate the added value and success of the framework or perform cost-benefit calculations. However, since the successful feasibility of the framework was visible and a portal concept was developed in the end that was positively evaluated by the sleep specialists, we refrained from such an additional procedure. A further limitation relates to the development of the framework for a ‘manageable’ project of a single institution (a university hospital in Germany). Unfortunately, no statements can be made about whether the framework is also suitable for more complex projects with several institutions, because it has not yet been applied for this. However, we think that the combination of methods is basically transferable, but the methods themselves might have to be modified again when used in multi-centre projects (e.g. development of several mockups/interface concepts adapted to the respective institution and its processes, interim evaluations with a larger number of sleep specialists and all participating institutions). With regard to the development of the portal, we can only find out how well the portal really works by conducting further testing in the real-world environment with the implemented system over an extended period of time. Currently, the project is not yet at this stage, but such evaluations are planned. This future work is intended to further complement our existing pragmatic framework. What we can take away from the last evaluation, however, is that sleep specialists rate the concept as positive. In this respect, we are on the right track.

## Conclusions

The paper presents a pragmatic methodological framework for the user-centred development of a portal to support patient management in a sleep laboratory and describes a set of (design and evaluation) methods that can be a valuable support for developers of comparable projects. We have gained very good experience with this combination in our project, where we had limited time and resources for concept development. For the future, we plan to implement the concept and test the portal in the clinical field and thus enrich our framework with additional methods.

## Supplemental Material

sj-docx-1-dhj-10.1177_20552076221134437 - Supplemental material for A pragmatic methodical framework for the user-centred development of an electronic process support for the sleep laboratory patients’ management Click here for additional data file. Supplemental material, sj-docx-1-dhj-10.1177_20552076221134437 for A pragmatic methodical framework for the user-centred development of an electronic process support for the sleep laboratory patients’ management by Maria Zerlik, Ian-C. Jung, Tony Sehr, Fabian Hennings, Christian Kamann, Moritz D. Brandt, Martin Sedlmayr and Brita Sedlmayr in Digital Health

sj-pptx-2-dhj-10.1177_20552076221134437 - Supplemental material for A pragmatic methodical framework for the user-centred development of an electronic process support for the sleep laboratory patients’ management Click here for additional data file. Supplemental material, sj-pptx-2-dhj-10.1177_20552076221134437 for A pragmatic methodical framework for the user-centred development of an electronic process support for the sleep laboratory patients’ management by Maria Zerlik, Ian-C. Jung, Tony Sehr, Fabian Hennings, Christian Kamann, Moritz D. Brandt, Martin Sedlmayr and Brita Sedlmayr in Digital Health

sj-docx-3-dhj-10.1177_20552076221134437 - Supplemental material for A pragmatic methodical framework for the user-centred development of an electronic process support for the sleep laboratory patients’ management Click here for additional data file. Supplemental material, sj-docx-3-dhj-10.1177_20552076221134437 for A pragmatic methodical framework for the user-centred development of an electronic process support for the sleep laboratory patients’ management by Maria Zerlik, Ian-C. Jung, Tony Sehr, Fabian Hennings, Christian Kamann, Moritz D. Brandt, Martin Sedlmayr and Brita Sedlmayr in Digital Health

sj-docx-4-dhj-10.1177_20552076221134437 - Supplemental material for A pragmatic methodical framework for the user-centred development of an electronic process support for the sleep laboratory patients’ management Click here for additional data file. Supplemental material, sj-docx-4-dhj-10.1177_20552076221134437 for A pragmatic methodical framework for the user-centred development of an electronic process support for the sleep laboratory patients’ management by Maria Zerlik, Ian-C. Jung, Tony Sehr, Fabian Hennings, Christian Kamann, Moritz D. Brandt, Martin Sedlmayr and Brita Sedlmayr in Digital Health

sj-pptx-5-dhj-10.1177_20552076221134437 - Supplemental material for A pragmatic methodical framework for the user-centred development of an electronic process support for the sleep laboratory patients’ management Click here for additional data file. Supplemental material, sj-pptx-5-dhj-10.1177_20552076221134437 for A pragmatic methodical framework for the user-centred development of an electronic process support for the sleep laboratory patients’ management by Maria Zerlik, Ian-C. Jung, Tony Sehr, Fabian Hennings, Christian Kamann, Moritz D. Brandt, Martin Sedlmayr and Brita Sedlmayr in Digital Health
