# Supplementary material for: A pragmatic methodical framework for the user-centred development of an electronic process support for the sleep laboratory patients’ management
Source: Digit Health. 2022 Oct 26;8:20552076221134437. doi: 10.1177/20552076221134437 (PMC9618751; doi:10.1177/20552076221134437)
Supplement: sj-docx-1-dhj-10.1177_20552076221134437 - Supplemental material for A pragmatic methodical framework for the user-centred development of an electronic process support for the sleep laboratory patients’ management [file sj-docx-1-dhj-10.1177_20552076221134437.docx]

**Appendix 1**. Key user characteristics of the three target groups. Explanation of abbreviations: Information source: SM=stakeholder meeting, OS=online search, H=hypotheses.

| Characteristic | User group 1:  (Specialist) physicians | User group 2:  Nurses | User group 3:  Patients |
| --- | --- | --- | --- |
| User group  [SM] | (specialist) physicians of the sleep laboratory and the sleep outpatient clinic of Dresden University Hospital | nurses of the sleep laboratory and the sleep ambulance of the University Hospital Dresden | patients with a sleep disorder who are receiving care at the Dresden University Hospital |
| Age range  [OS] | assumed average age: 40-59 years  (according to 2019 statistics, the majority of inpatient physicians are between 40-59 years old) | assumed average age: 45-54 years (46% of registered nurses in general hospitals with 100 beds or more is 45 years and older); increasing proportion of older nurses over the next few years | variable - depending on sleep disorder: e.g. insomnia: highest prevalence in patients aged 40-59 years; sleep apnoea: highest prevalence in patients aged 55+ |
| Gender  [OS] | overall more male (specialist) internists (74.3%) and more male neurologists (58.4%) than female (specialist) internists and neurologists | assume a higher proportion of female nurses, according to 2019 statistics, the proportion of female nurses was about 80% | variable - depending on sleep disorder: e.g. insomnia: women are significantly more often  affected than men; sleep apnoea: women are ten times less likely to be affected |
| Language and culture [OS] | German will be the main language; however, there will also be foreign physicians with limited knowledge of German (increase in the proportion of foreign physicians working in Germany) | German will be the main language; however, there are also an increasing number of foreign nursing staff with limited knowledge of German (in the nursing sector, the proportion of foreign employees was eight percent) | German will be the main language; however, there are also patients with a migration background and limited knowledge of German |
| Work experience / qualifications [OS] | each level- from less to highly experienced (specialist) physicians (on average 14 years of professional experience/ highly qualified) | each level- from less to highly experienced nurses each educational group- from less to highly educated patients | each educational group- from less to highly educated patients |
| Physical limitations [OS] | include physicians with visual impairments (e.g. ametropia, red/green blindness) | include nurses with visual impairments (e.g. ametropia, red/green blindness) | include patients with visual impairments (e.g. ametropia, red/green blindness) |
| Previous experience with similar systems [H] | previous experience with an e-health portal/telemedicine portal usually does not exist (since no such system is currently available) | previous experience with an e-health portal/telemedicine portal usually does not exist (since no such system is currently available) | previous experience with e-health portals/telemedicine portals does not usually exist |
| Computer experience [OS] | variable-depending on system and application; assume intermediate computer experience | variable-depending on system and application; assume intermediate computer experience | variable-depending on system and application; assume intermediate computer experience |
| Task knowledge  [SM] | variable- depending on work experience; assume good task knowledge | variable- depending on work experience; assume good task knowledge | variable- depending on previous duration of sleep disorder; assume low task knowledge |
| Previous training  [SM] | there is no training planned for the use of the e-health portal, therefore do not assume any training | there is no training planned for the use of the e-health portal, therefore do not assume any training | there is no training planned for the use of the e-health portal, therefore do not assume any training |
| Frequency of use  [H] | initially only first-time users, since new application; later variable: first-time users (new staff/rotation staff) or routine users; usage regularly for patient management) | initially only first-time user, as new application; later variable: first-time user (new staff) or routine user; usage regularly for patient management) | initially only first-time users, since new application; later variable: first-time user or occasional user |
| Motivations for use  [SM] | implementation of a telemedical care process and improvement of patient management; assume a rather high motivation to use the system | implementation of a telemedical care process and improvement of patient management; assume a rather high motivation to use the system | variable - depending on type of sleep disorder and attitude towards telemedicine (acceptance); assume medium motivation for use |
| Fears, concerns about the system  [OS] | complexity of the system, lack of integration into the workflow, time required for use, user-friendliness/usability, errors during use (e.g. incorrect entries) | complexity of the system, lack of integration into the workflow, time required for use, user-friendliness/usability, errors during use (e.g. incorrect entries) | poor usability/difficulty in using the system; fear of making mistakes (low self-efficacy expectation); no recognisable added value/additional benefit of the portal; fear of lack of data protection |
