# Supplementary figures and images for: A pragmatic methodical framework for the user-centred development of an electronic process support for the sleep laboratory patients’ management
Source: Digit Health. 2022 Oct 26;8:20552076221134437. doi: 10.1177/20552076221134437 (PMC9618751; doi:10.1177/20552076221134437)

## Slide 1
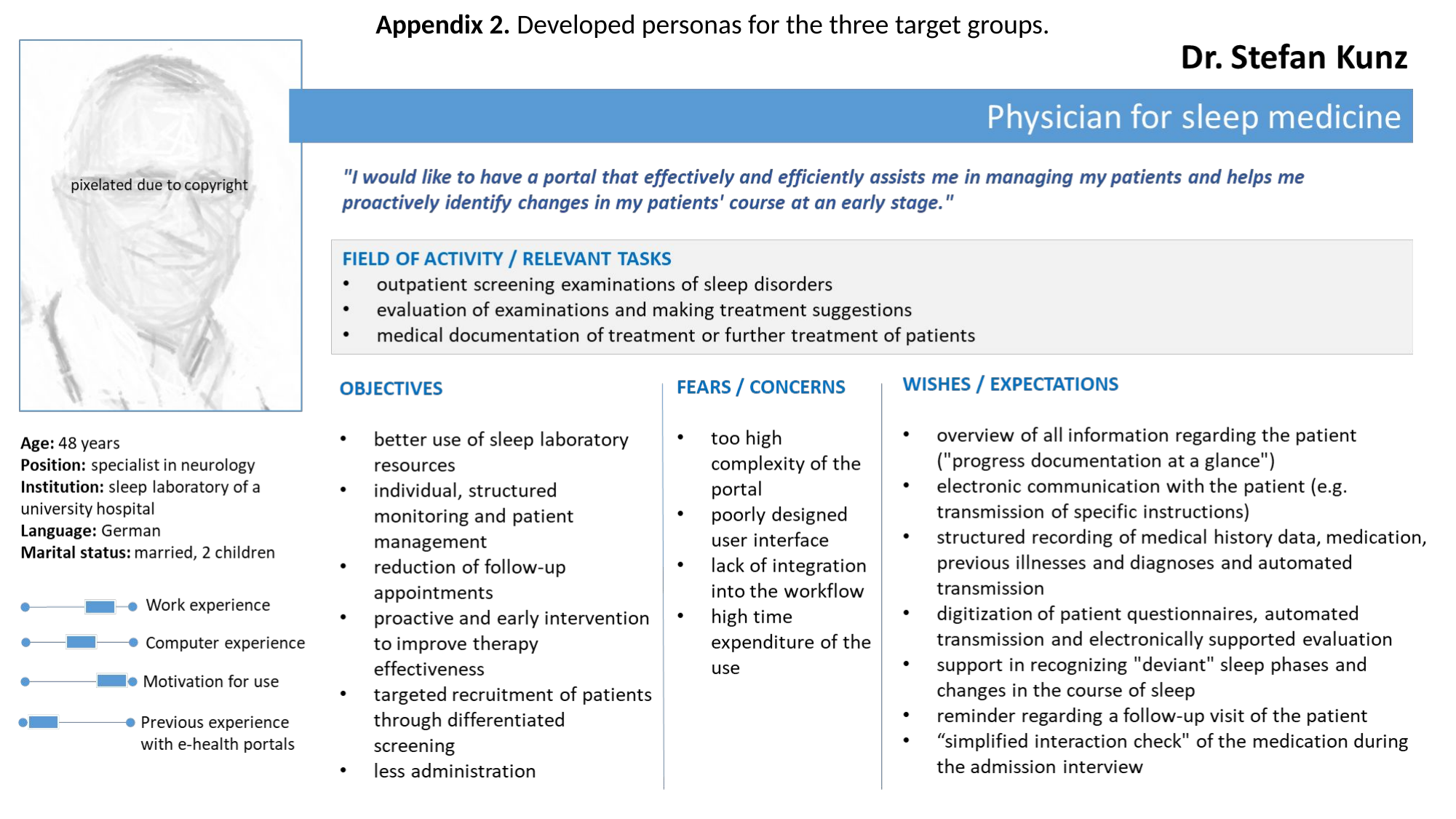

Appendix 2. Developed personas for the three target groups.

## Slide 2
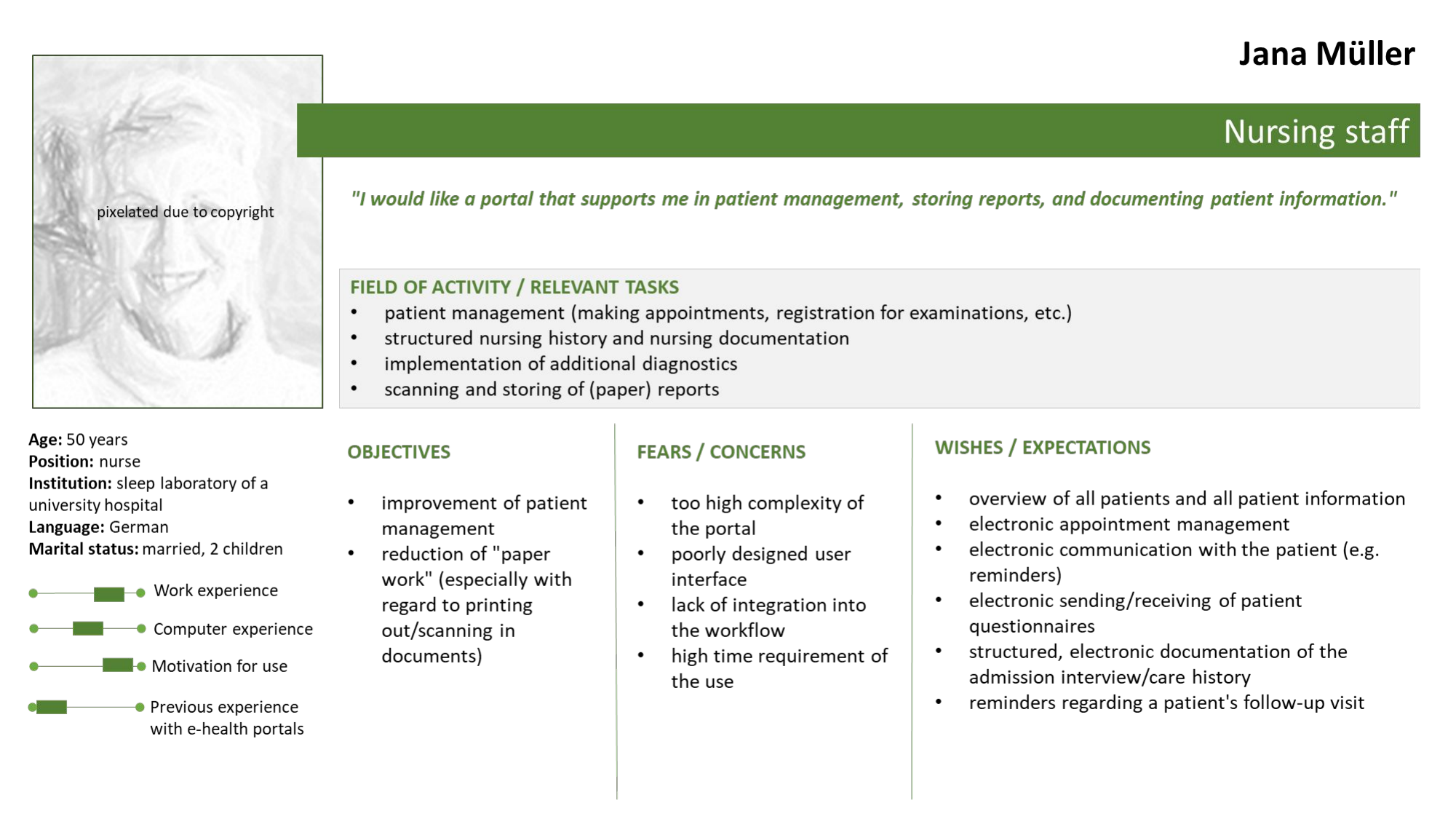

## Slide 3
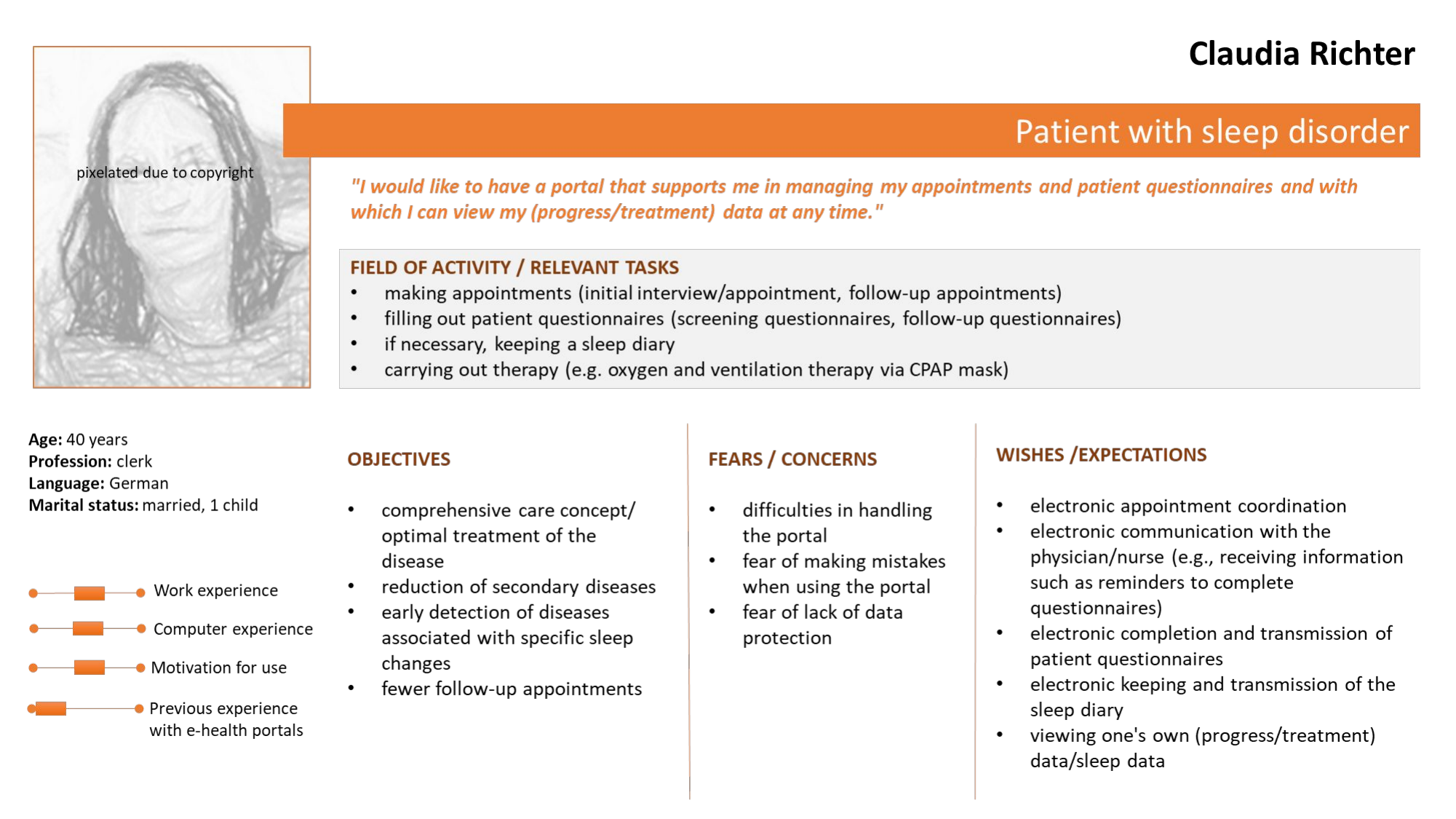

Supplement: sj-pptx-2-dhj-10.1177_20552076221134437 - Supplemental material for A pragmatic methodical framework for the user-centred development of an electronic process support for the sleep laboratory patients’ management [file sj-pptx-2-dhj-10.1177_20552076221134437.pptx]
