# Supplementary material for: A pragmatic methodical framework for the user-centred development of an electronic process support for the sleep laboratory patients’ management
Source: Digit Health. 2022 Oct 26;8:20552076221134437. doi: 10.1177/20552076221134437 (PMC9618751; doi:10.1177/20552076221134437)
Supplement: sj-docx-3-dhj-10.1177_20552076221134437 - Supplemental material for A pragmatic methodical framework for the user-centred development of an electronic process support for the sleep laboratory patients’ management [file sj-docx-3-dhj-10.1177_20552076221134437.docx]

**Appendix 3.** List of the highest prioritised requirements (priority 1: absolutely to be implemented in the project).

| **Tasks to be supported by the portal** | **Derived usage requirements** |
| --- | --- |
| **user group 1: physician** |  |
| prepare the medical history | - The user must be able to obtain information about the patient in the portal (based on information already entered, e.g. questionnaires on sleep disorders). - The user must be able to compile/select information (e.g., symptoms) to be queried in the portal for the case history (=supported case history creation). |
| document the medical history interview | - The user must be able to document the anamnesis interview in the portal in a structured manner/according to a fixed structure. - The user must be able to make free notes/additions to individual aspects in the portal. - The user must receive a summary of the documented anamnesis data in the portal in order to be able to transfer it manually (copy & paste) to ORBIS in the physician's letter. |
| interpret sleep records and make a diagnosis/evaluate the success of therapy | - The user must be able to view the results of the sleep recording and other information of the patient in the portal. |
| document decisions/diagnoses, recommendations/therapies and orders | - The user must be able to document decisions/diagnoses, recommendations/therapies and orders in the portal. |
| write a (provisional) doctor's letter, if necessary write an aid prescription | - The user must be able to obtain a summary of the previously documented diagnoses, recommendations and therapies in the portal in order to be able to transfer them manually (copy & paste) to ORBIS in letters/orders. |
| review ResMed respiratory mask data (breathing interruptions, usage problems, etc.) | - The user must be able to view ResMed respiratory mask data in the portal. |
| schedule patient for reappearance/order follow-up | - The user must be notified in the portal of upcoming appointments for the patient, depending on the previously entered sleep diagnosis. |
| conduct progress control | - The user must be able to quickly and easily compare current findings with previous results in the portal. |
| **user group 2: nurse** |  |
| register patient in the portal | - The user must be able to enter a patient in a patient list in the portal. - The user must be able to view the referral documents scanned into ORBIS for the registered patient in the portal. |
| review admission/ nursing anamnesis data | - The user must be able to view documented nursing history information in the portal. |
| hand over questionnaires on sleep history and sleep disorders to the patient on site | - The user must be able to provide access to sleep history and sleep disorder questionnaires for the patient in the portal. |
| **user group 3: patient** |  |
| complete questionnaires (medical history/sleep questionnaires) or a sleep diary | - The user must be able to access certain questionnaires/sleep diary in the portal. - The user must be able to fill in certain questionnaires/sleep diary electronically in the portal. - The user must be able to save all entries made in the portal/store the completed questionnaires/sleep diary in the portal. |
| upload findings of outpatient practitioners to the portal | - The user must be able to upload/store findings from outpatient practitioners in the portal. |
| view own data (findings, sleep data, progress/trends) | - The user must be able to view his/her own sleep data, progressions (e.g. questionnaire scores) in the portal. - The user must be able to recognize improvements or deteriorations of progressions in the portal. |
