# Supplementary material for: A pragmatic methodical framework for the user-centred development of an electronic process support for the sleep laboratory patients’ management
Source: Digit Health. 2022 Oct 26;8:20552076221134437. doi: 10.1177/20552076221134437 (PMC9618751; doi:10.1177/20552076221134437)
Supplement: sj-docx-4-dhj-10.1177_20552076221134437 - Supplemental material for A pragmatic methodical framework for the user-centred development of an electronic process support for the sleep laboratory patients’ management [file sj-docx-4-dhj-10.1177_20552076221134437.docx]

| Guidelines for… | Relevant Topics | Reference |
| --- | --- | --- |
| EMR and EHR Interfaces (focused on the diagnosis process and documentation) | - Naturalness - Consistency - Preventing errors - Minimising cognitive overload - Efficient interaction - Forgiveness and feedback - Effective use of language - Effective information presentation - Customisation / flexibility - Safety issues | Zahabi M, Kaber DB, Swangnetr M. Usability and Safety in Electronic Medical Records Interface Design: A Review of Recent Literature and Guideline Formulation. Hum Factors. 2015 Aug;57(5):805-34. doi: 10.1177/0018720815576827. Epub 2015 Mar 23. PMID: 25850118. |
| Visual Information Display | - Arrangement and labelling of information - Lists - Tables - Coding methods (graphical coding) | International Organization for Standardization. DIN EN ISO 9241-125:2018-08. Ergonomics of human-system interaction - Part 125: Guidance on visual presentation of information (ISO 9241-125:2017); German version EN ISO 9241-125:2017. |
| Form Dialogs | - Layout - Navigation and scrolling - Interaction with the form - Form elements | International Organization for Standardization. DIN EN ISO 9241-143:2012-06. Ergonomics of human-system interaction - Part 143: Forms (ISO 9241-143:2012); German version EN ISO 9241-143:2012. |
| Visual User Interface Elements | - Description of when and how to use user interface elements:   - Structuring elements (drop-down, menu, pop-up)   - Layout elements (tabs, window)   - Interaction elements (slider, toolbar, tool-tip) | International Organization for Standardization. DIN EN ISO 9241-161:2016-10. Ergonomics of human-system interaction - Part 161: Guidance on visual user-interface elements (ISO 9241-161:2016); German version EN ISO 9241-161:2016. |
| User Interfaces for the World Wide Web | - Content concept - Navigation - Search - Presentation of content - General design aspects | International Organization for Standardization. DIN EN ISO 9241-151:2008-09. Ergonomics of human-system interaction - Part 151: Guidance on World Wide Web user interfaces (ISO 9241-151:2008); German version EN ISO 9241-151:2008 |
| User-Oriented Security for Telehealth Services & Data Privacy Aspects | - Requirements for telehealth services to maximize the perception of security and privacy by users | Vega-Barbas M, Seoane F, Pau I. Characterization of User-Centered Security in Telehealth Services. Int J Environ Res Public Health. 2019 Feb 26;16(5):693. doi: 10.3390/ijerph16050693. PMID: 30813642; PMCID: PMC6427719. |

**Appendix 4.** Usability guideline collection.
